# Supplementary material for: Chemical Compounds of Berry-Derived Polyphenols and Their Effects on Gut Microbiota, Inflammation, and Cancer
Source: Molecules. 2022 May 20;27(10):3286. doi: 10.3390/molecules27103286 (PMC9146061; doi:10.3390/molecules27103286)
Supplement: Supplementary file 1 [file molecules-27-03286-s001.zip › molecules-1679146-supplementary.pdf]

molecules MDPI

Review

Chemical Compounds of Berry-Derived Polyphenols and Their Effects on Gut Microbiota, Inflammation, and Cancer

Abdelhakim Bouyahya [1,*], Nasreddine El Omari [2], Naoufal EL Hachlafi [3], Meryem El Jemly [4], Maryam Hakkour [5], Abdelaali Balahbib [5], Naoual El Menyiy [6], Saad Bakrim [7], Hanae Naceiri Mrabti [8], Aya Khouchlaa [9], Mohamad Fawzi Mahomoodally [10], Michelina Catauro [11,*], Domenico Montesano [12] and Gokhan Zengin [13,*]

1 Laboratory of Human Pathologies Biology, Department of Biology, Faculty of Sciences, Mohammed V University in Rabat, Rabat 10106, Morocco
2 Laboratory of Histology, Embryology, and Cytogenetic, Faculty of Medicine and Pharmacy,
Mohammed V University in Rabat, Rabat 10100, Morocco; nasrelomari@gmail.com
3 Microbial Biotechnology and Bioactive Molecules Laboratory, Sciences and Technologies Faculty, Sidi Mohmed Ben Abdellah University,
Imouzzer Road Fez, Fez B.P. 1796, Morocco; naoufal.elhachlafi@usmba.ac.ma
4 Faculty of Pharmacy, University Mohammed VI for Health Science, Casablanca 82403, Morocco;
eljemli.meryem@gmail.com
5 Laboratory of Biodiversity, Ecology, and Genome, Faculty of Sciences, Mohammed V University in Rabat, Rabat 10106, Morocco; maryam.hakkour@gmail.com (M.H.), balahbib.abdo@gmail.com (A.B.)
6 Laboratory of Pharmacology, National Agency of Medicinal and Aromatic Plants, Taounate 34025, Morocco; Nawal.ELMENYIY@usmba.ac.ma
7 Molecular Engineering, Valorization and Environment Team, Polydisciplinary Faculty of Taroudant,
Ibn Zohr University, Agadir 80000, Morocco; s.bakrim@hotmail.com
8 Laboratory of Pharmacology and Toxicology, Bio Pharmaceutical and Toxicological Analysis Research Team, Faculty of Medicine and Pharmacy, Mohammed V University, Rabat BP 6203, Morocco; naceiri.mrabti.hanae@gmail.com
9 Laboratory of Biochemistry, National Agency of Medicinal and Aromatic Plants, Taounate 34025, Morocco; aya.khouchlaa@gmail.com
10 Department of Health Sciences, Faculty of Medicine and Health Sciences, University of Mauritius, Reduit 80837, Mauritius; f.mahomoodally@uom.ac.mu
11 Department of Engineering, University of Campania "Luigi Vanvitelli", Via Roma 29, 81031 Aversa, Italy
12 Department of Pharmacy, University of Naples Federico II, Via D. Montesano 49, 80131 Naples, Italy;
domenico.montesano@unina.it
13 Physiology and Biochemistry Research Laboratory, Department of Biology, Science Faculty,
Selcuk University, Konya, Turkey
* Correspondence: boyahyaa-90@hotmail.fr (A.B.); michelina.catauro@unicampania.it (M.C.);
gokhanzengin@selcuk.edu.tr (G.Z.)

Citation: Bouyahya, A.; Omari, N.E.; Hachlafi, N.E.; Jemly, M.E.; Hakkour, M.; Balahbib, A.; Menyiy, N.E.; Bakrim, S.; Mrabti, H.N.; Khouchlaa, A.; et al. Chemical Compounds of Berry-Derived Polyphenols and Their Effects on Gut Microbiota, Inflammation, and Cancer. *Molecules* **2022**, *27*, 3286. https://doi.org/10.3390/molecules27103286

Academic Editor: Francesco Cacciola

Received: 28 March 2022
Accepted: 8 May 2022
Published: 20 May 2022

Publisher's Note: MDPI stays neutral with regard to jurisdictional claims in published maps and institutional affiliations.

Copyright: © 2022 by the authors. Submitted for possible open access publication under the terms and conditions of the Creative Commons Attribution (CC BY) license (https://creativecommons.org/licenses/by/4.0/).

molecules MDPI

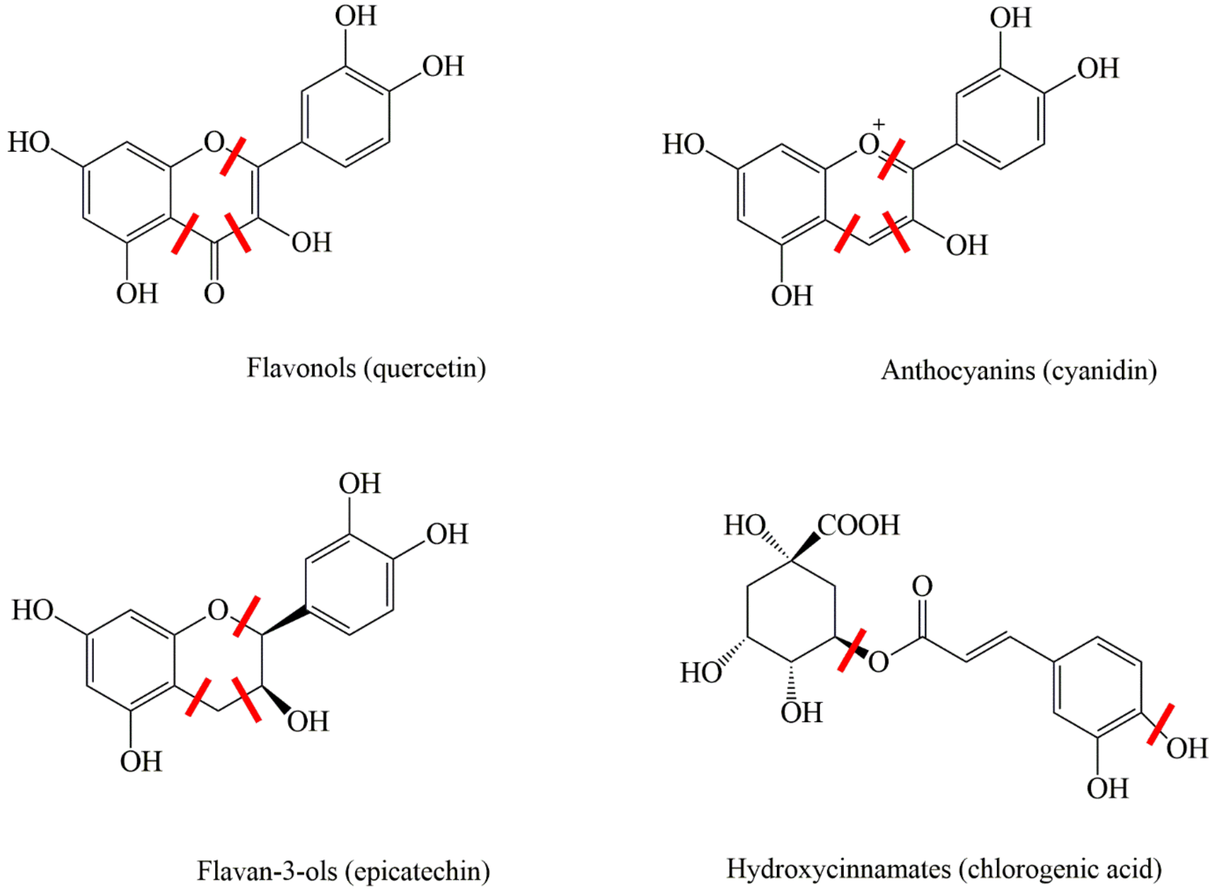

Figure S1:Gut microbiota heterocyclic C ring cleavage of berries polyphenols. (▬) Positions of the potential C-ring cleavages. (Adapted from Selma et al., 2009)

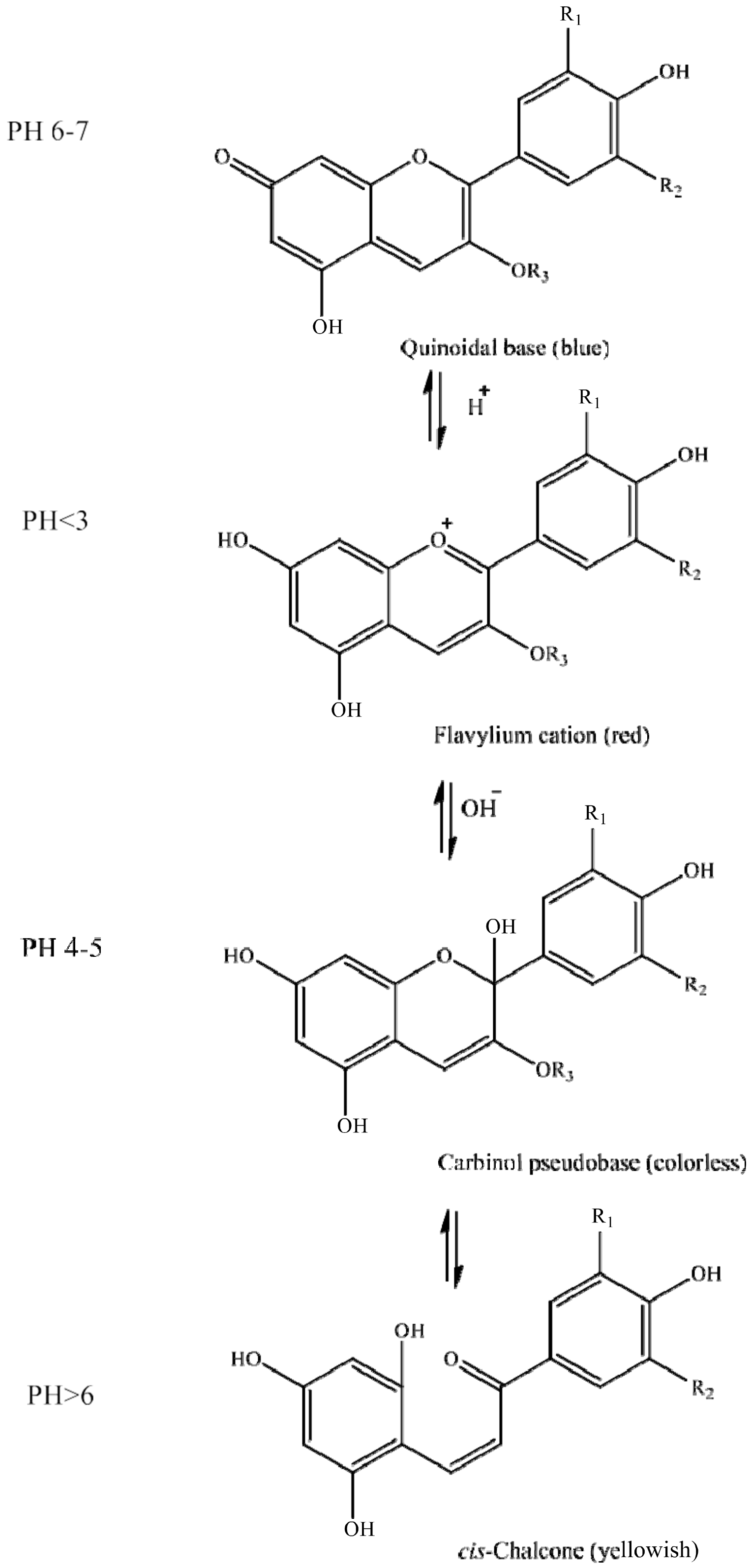

Figure S2: Molecular structure of berry anthocyanins under different pH conditions.
